# Immune Alterations in Patients with Anti-Interferon-γ Autoantibodies

**DOI:** 10.1371/journal.pone.0145983

**Published:** 2016-01-04

**Authors:** Nuttapol Chruewkamlow, Kodchakorn Mahasongkram, Supansa Pata, Romanee Chaiwarith, Parichart Salee, Khuanchai Supparatpinyo, Watchara Kasinrerk

**Affiliations:** 1 Biomedical Technology Research Center, National Center for Genetic Engineering and Biotechnology, National Sciences and Technology Development Agency at the Faculty of Associated Medical Sciences, Chiang Mai University, Chiang Mai, Thailand; 2 Department of Medical Technology, Division of Clinical Immunology, Faculty of Associated Medical Sciences, Chiang Mai University, Chiang Mai, Thailand; 3 Department of Internal Medicine, Faculty of Medicine, Chiang Mai University, Chiang Mai, Thailand; 4 Research Institute for Health Sciences, Chiang Mai University, Chiang Mai, Thailand; University of Thessaly, Faculty of Medicine, GREECE

## Abstract

Autoantibodies against interferon-gamma (IFN-γ) can cause immunodeficiency and are associated with various opportunistic infections. In the present study, we investigated other cellular immune parameters for a better understanding of the immunodeficiency condition in the patients. The numbers of WBC, monocytes and NK cells were increased in patients with anti-IFN-γ autoantibodies (AAbs). Upon TCR activation, T cell proliferation and IL-2 receptor of the patients remained intact. Nonetheless, the Th1 cytokine (IFN-γ and TNF-α) production was up-regulated. The production of Th2 (IL-4) and Th17 (IL-17) cytokines was unchanged. We suggest that, in addition to the presence of anti-IFN-γ autoantibodies, alterations in the cellular immune functions may also contribute to this immunodeficiency.

## Introduction

Immune system plays an important role in the eradication of infectious diseases and cancers. Immunodeficiency, caused by either genetic defects or infections, leads to an increased predisposition to infections and malignancy. In recent years, autoantibodies (AAbs) against cytokines in humans have been reported [1–7]. These anti-cytokine AAbs can neutralize host cytokines and disrupt the immune function causing pathogenesis and immunodeficiency. Anti-granulocyte macrophage colony stimulating factor (GM-CSF) AAbs can cause pulmonary alveolar proteinosis (PAP) [8]. Anti-IL-1α, IL-12 and TNF-α AAbs can be found in SLE patients [9]. The presence of anti-IL-17 or anti-IL-22 AAbs in patients was associated with chronic mucocutaneous candidiasis [6, 10]. Moreover, anti-IFN-γ AAbs was shown to be associated with immunodeficiency [1–5, 11, 12].

IFN-γ is a critical cytokine involved in immune responses and is produced by various cells, including natural killer (NK) cells, natural killer T (NKT) cells, CD4^+^ T helper 1 (Th1) cells, and CD8^+^ T cells [13–17]. Depletion of IFN-γ production is associated with abnormalities of both the innate and adaptive immune responses and, therefore, increases the susceptibility to infection, particularly intracellular microbes [18–20]. During the last decade, immunodeficiency due to anti-IFN-γ AAbs has been described [1–4, 11, 12, 21]. Autoantibodies against IFN-γ have been reported to exist in the serum of these patients [1–4, 11, 21]. The anti-IFN-γ AAbs titer was significantly higher among the patients with active opportunistic infections compared to those without opportunistic infections [1, 4]. Interferon receptor 1 expression on patients’ lymphocytes, however, was fundamentally normal [1]. The anti-IFN-γ AAbs was shown to neutralize IFN-γ activity in the body and lead to immunodeficiency [1]. The mechanism initiating the production of anti-IFN-γ AAbs, however, remains unknown.

Severe or disseminated non-tuberculous mycobacteria and other opportunistic infections are regularly observed in patients with anti-IFN-γ AAb [1–4, 11, 21]. In addition to the presence of anti-IFN-γ AAbs, we raise the question whether other immune-abnormalities also contribute to the immunodeficiency in these patients. To address this question, in this study, the numbers of phagocytes and lymphocyte sub-population involved in the immune responses, such as T cells sub-populations, B cells, NK cells, and NKT cells were determined. Additionally, T cell activation and function were investigated. The results obtained may lead to a better understanding of the immunodeficiency condition of the patients.

## Materials and Methods

### Study population

Patients with immunodeficiency and anti-IFN-γ AAbs were diagnosed at Maharaj Nakorn Chiang Mai Hospital, Chiang Mai University. All patients were anti-IFN-γ AAbs positive and HIV negative. Healthy normal subjects were included as a control group. All the healthy subjects were anti-IFN-γ AAbs negative. The determination of anti-IFN-γ AAbs in plasma or sera was carried out using an indirect ELISA, as previously reported [4, 12].

In this study, 36 patients (18 female and 18 male) with anti-IFN-γ AAbs were enrolled and their average age was 54 years old (range: 37 to 77). Ten healthy subjects (8 female and 2 male) were also recruited and their average age was 26 years old (range: 23 to 32).

The study was approved by the ethics committees of the Faculty of Medicine and the Research Institute for Health Sciences at Chiang Mai University. Written informed consent was obtained for each subject prior to enrollment.

### Leukocyte distribution analysis

Blood samples were collected from subjects in tubes containing acid citrate dextrose as the anti-coagulant. Leukocyte distributions in the patients with anti-IFN-γ AAbs and the healthy subjects were determined by flow cytometry in combination with complete blood count (CBC) data. Lymphocyte sub-populations were determined by the lysed whole blood staining method, using the following antibodies: (i) PerCP conjugated anti-CD45, PE conjugated anti-CD4, and FITC conjugated anti-CD3 monoclonal antibodies (mAbs) (BD Biosciences, San Jose, CA, USA) were used for the enumeration of CD4^+^ T lymphocytes; (ii) PerCP conjugated anti-CD45, PE conjugated anti-CD8, and FITC conjugated anti-CD3 mAbs (BD Biosciences) were used for the enumeration of CD8^+^ T lymphocytes; (iii) PerCP conjugated anti-CD45, PE conjugated anti-CD56, and anti-CD16 mAbs were used for the enumeration of NK cells; (iv) FITC conjugated anti-CD3 mAb was used in combination with PerCP conjugated anti-CD45, PE conjugated anti-CD56, and anti-CD16 mAbs for counting NKT cells; (v) FITC conjugated anti-CD45, PE conjugated anti-CD14 (BD Biosciences), and PerCP conjugated anti-CD19 mAbs (BioLegend, San Diego, CA, USA) were used for the enumeration of CD19^+^ B lymphocytes.

For the staining method, 50 μL of whole blood was stained with 10 μL of the appropriate combination of mAbs for 30 min at room temperature. The red blood cells were then lysed using FACS lysing solution (BD Biosciences). The cells were then washed twice with a washing reagent (1% fetal bovine serum [FBS], 0.02% NaN_3_ in phosphate buffered saline [PBS]) and analyzed using a FACSort flow cytometer (BD Biosciences). For flow cytometric analysis, the lymphocyte population was gated using CD45 expression and side scatter signals. The cell sub-populations were then determined according to their surface markers expression.

### CFSE-based proliferation assay

Peripheral blood mononuclear cells (PBMCs) were separated from blood by gradient centrifugation over Ficoll-Hypaque solution (GE Healthcare Life Sciences, Pittsburgh, PA, USA). PBMCs (1×10^7^ cells/mL) were incubated with carboxyfluorescein succinimidyl ester (CFSE; Invitrogen/Molecular Probes, Eugene, OR, USA) at a final concentration of 0.5 μM for 10 min at 37°C. The cells were then washed with 10% FBS in RPMI two times in order to remove any excessive CFSE. The CFSE-labeled PBMCs (5×10^5^ cells/mL) were stimulated with or without immobilized anti-CD3 mAb clone OKT3 (Ortho Pharmaceuticals, Raritan, NJ, USA) (60 ng/mL) for 3 days in a 5% CO_2_ incubator at 37°C. The cells were harvested and investigated for cell proliferation by monitoring the reduction in CFSE using FACSort flow cytometer (BD Biosciences).

### CD25 (Interleukin-2 receptor) determination

PBMCs were activated with or without anti-CD3 mAb OKT3 (Ortho Pharmaceuticals), as was described above. On the third day of cultivation, the cells were harvested and stained for CD25 expression using FITC-conjugated anti-CD25 mAb (Beckman Coulter, Marseille, France). The expression of CD25 in lymphocytes was assessed by FACSort flow cytometer (BD Biosciences).

### Intracellular cytokine staining

To determine the cytokine production of T cells, PBMCs were activated using 10 ng/mL phorbolmyristate acetate (PMA; Sigma-Aldrich, MO, USA) and 1μg/mL ionomycin (Sigma-Aldrich) in the presence of 5 μg/mL Brefeldin A (Sigma-Aldrich) for 6 h. The cells were then harvested, washed once with washing reagent (1% FBS, 0.02% NaN_3_ in PBS), and stained with PerCP conjugated anti-CD3 mAb for 30 min at 4°C. The stained cells were fixed using 200 μL fixation buffer (4% paraformaldehyde in PBS) for 20 min at RT. The fixed cells were then washed twice with PBS and once with permeabilization buffer (5% FBS, 0.1% saponin, 0.02% NaN_3_ in PBS), and then incubated for 15 min at room temperature. The intracellular cytokine was stained with PE conjugated anti-IFN-γ, anti-IL-4, anti-IL-17A, or anti-TNF-α mAb for 30 min at 4°C. After that, the stained cells were washed with permeabilization buffer and re-suspended in staining buffer, following which the cells were subjected to flow cytometer (FACSort, BD Biosciences) and analyzed with the FlowJo software (Tree Star, Inc. Ashland, USA). All the PE conjugated anti-cytokine mAbs were purchased from BioLegend (San Diego, CA, USA).

For the flow cytometric analysis, the T cells were gated according to the expression of the PerCP conjugated anti-CD3 mAb. The gated CD3^+^ T cells were further assessed for intracellular cytokine-producing cells by monitoring the PE-positive cells.

### Statistical analysis

The significance of the difference between the compared populations was analyzed by using the Mann-Whitney U test. A *p* value less than *0*.*05* was considered significant.

## Results

### Leukocyte distribution in patients with anti-IFN-γ AAbs

Peripheral blood leukocyte distribution was compared between the patients with anti-IFN-γ AAbs and healthy subjects. The total number of white blood cells was significantly higher in the patients with anti-IFN-γ AAbs than the healthy subjects (Table 1). In addition, the monocyte count was also found to be higher in the patients with anti-IFN-γ AAbs patients (Table 1). However, there was no difference in the absolute lymphocyte counts between tested groups (Table 1).

**Table 1:** Leukocyte Distribution in the patients with anti-IFN-γ Autoantibodies and Healthy Subjects.

	Patients with anti-IFN-γ AAbs (N = 29)	Healthy subjects (N = 10)	*p*-value*
**WBC (×10**^**3**^ **cells/μL)**	8.9±4.3	6.2±1.0	***<0*.*05***
**Neutrophil (×10**^**3**^ **cells/μL)**	5.5±3.8	3.6±0.9	*0*.*160*
**Monocyte (×10**^**2**^ **cells/μL)**	5.3±1.4	3.9±0.9	***<0*.*005***
**Lymphocyte (×10**^**2**^ **cells/μL)**	22.2±10.9	35.8±8.9	*0*.*857*
**CD3**^**+**^**T cell (×10**^**2**^ **cells/μL)**	12.9±6.2	14.1±3.8	*0*.*258*
**CD4**^**+**^**T cell (×10**^**2**^ **cells/μL)**	6.5±3.4	7.8±2.2	*0*.*092*
**CD8**^**+**^**T cell (×10**^**2**^ **cells/μL)**	5.6±2.9	6.3±2.5	*0*.*290*
**CD19**^**+**^**cell (×10**^**2**^ **cells/μL)**	3.2±2.0	3.9±1.7	*0*.*087*
**CD3**^**-**^**CD56**^**+**^ **NK cells (×10**^**2**^ **cells/μL)**	4.6±2.0	2.8±1.7	***<0*.*05***
**CD3**^**+**^**CD56**^**+**^ **NKT cells (cells/μL)**	98±77	126±75	*0*.*126*

*Comparison between patients with anti-IFN-γ AAbs and healthy subjects. Boldfacing indicates statistical significance.

The lymphocyte sub-populations were also determined by staining with specific set of mAbs. By flow cytometric analysis, lymphocytes were gated according to the CD45 expression and their size and the percentages of lymphocyte sub-populations were determined. We found that the total numbers of the CD3^+^, CD4^+^, CD8^+^ T and CD19^+^ B cells were not significantly changed (Table 1). The NK (CD3^-^ CD56^+^) cell population was increased in the patients with anti-IFN-γ AAbs.

### T cell proliferation and CD25 expression of the patients with anti-IFN-γ AAbs

The ability of T cells activation in the patients with anti-IFN-γ AAbs was investigated using the CFSE based proliferation assay. Upon CD3 stimulation, the percentage of divided cells was not significantly different between patients with anti-IFN-γ AAbs and the healthy subjects (S1 Fig).

We also investigated the expression of CD25 (IL-2 receptor) of the CD3-activated lymphocytes. The lymphocytes were gated and assessed for the expression of CD25 using FITC-conjugated anti-CD25 mAb (S2A Fig). The mean fluorescence intensity (MFI) ratio of the CD25 expression (MFI of stimulation/MFI of un-stimulation) (S2B Fig) and the percentage of the CD25 expressing cells (S2C Fig) were not significantly different between the two groups.

### Cytokine production of T cells of the patients with anti-IFN-γ AAbs

**Fig 1:**
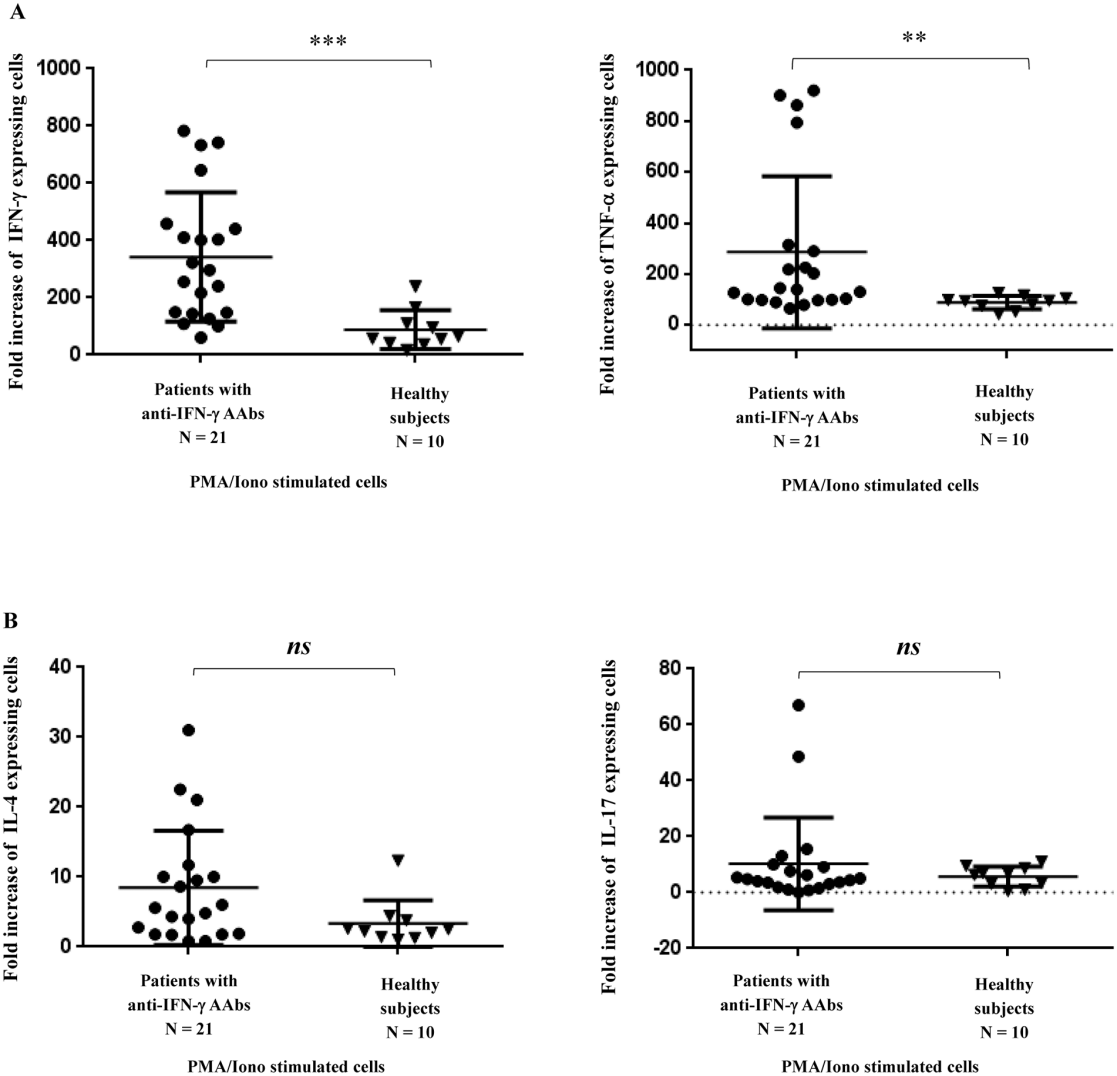
Intracellular cytokine production of patients with anti-IFN-γ AAbs and healthy subjects. PBMCs were stimulated with PMA and ionomycin (Iono). The PBMCs were then stained surface CD3 using PerCP conjugated anti-CD3 mAb and PE conjugated anti-cytokine antibody. The expression of the indicated intracellular cytokines of the CD3^+^ T cell was analyzed by flow cytometry. The fold increase in the cytokine production in response to the stimulants as compared to cell culture with no stimulants is shown. “*ns* represents no statistical significance”; “**” represents *p<0*.*005*; “***” represents *p<0*.*001*.

In order to assess the function of T cells in the patients with anti-IFN-γ AAbs, we determined the production of various intracellular cytokines upon T cell activation. After PMA and ionomycin stimulation, the Th1 cytokine production, including IFN-γ and TNF-α, were significantly up-regulated in the patients with anti-IFN-γ AAbs in comparison with the healthy subjects (Fig 1A). However, the Th2 and the Th17 cytokines, IL-4 and IL-17, in the patients were not statistically significantly different from the healthy subjects (Fig 1B).

**Fig 2:**
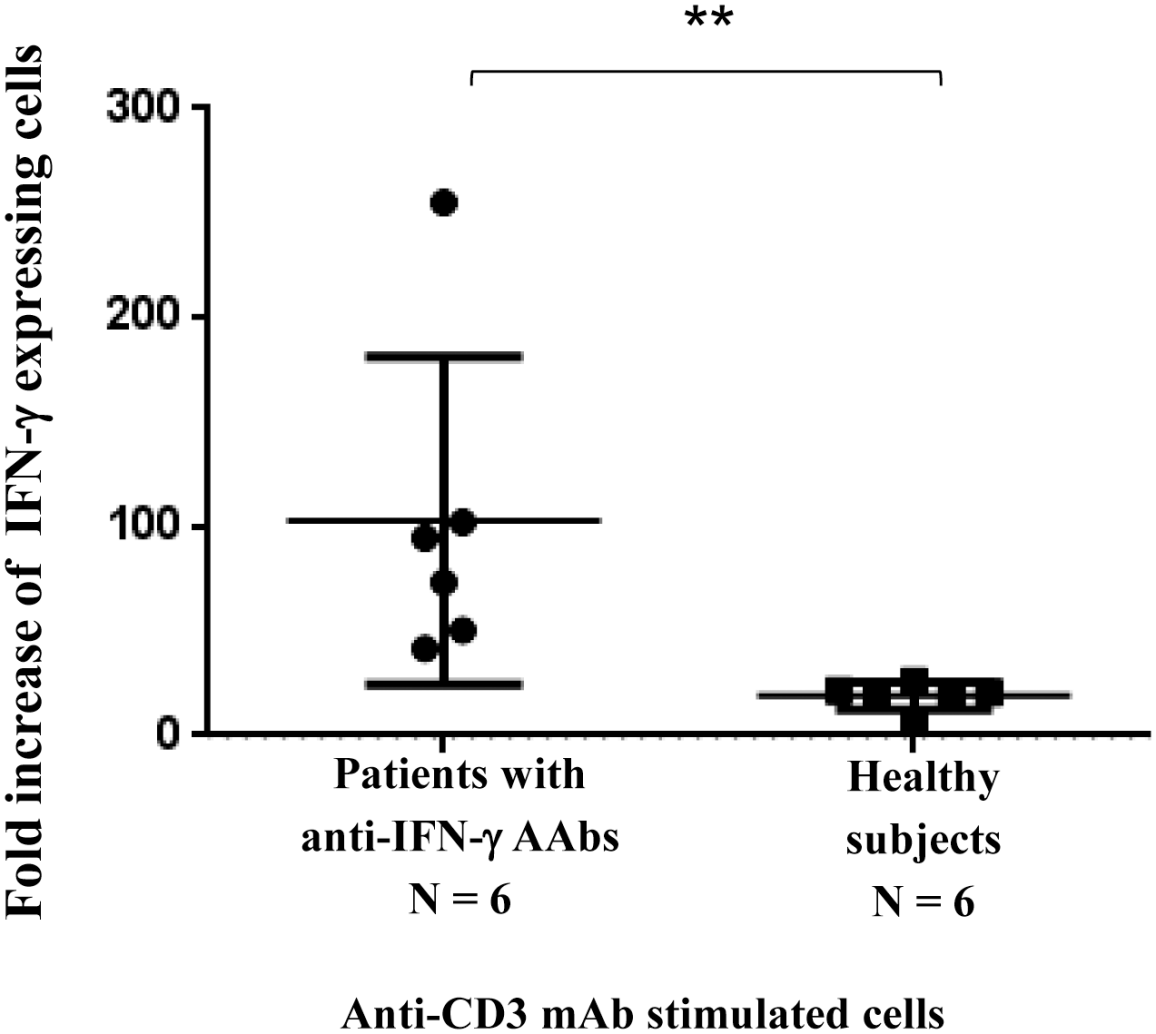
Intracellular interferon-γ production in patients with anti-IFN-γ AAbs and healthy subjects. PBMCs were stimulated with immobilized anti-CD3 mAb. The PBMCs were then stained surface CD3 using PerCP conjugated anti-CD3 mAb and PE conjugated anti-cytokine antibody. The expression of the intracellular IFN-γ of the CD3^+^ T cell was analyzed by flow cytometry. The fold increase in the IFN-γ production in response to the stimulants as compared to cell culture with no stimulants is shown. “**” represents *p<0*.*005*.

Stimulation of T cells by anti-CD3 mAb was performed. The IFN-γ production in the patients with anti-IFN-γ AAbs was significantly up-regulated compared to the heathy subjects (Fig 2). Our results simply reflect an alteration of the cell-mediated immune response in patients with anti-IFN-γ AAbs.

## Discussion

The presence of anti-IFN-γ autoantibodies has been demonstrated to be strongly associated with immunodeficiency syndrome in adults, leading to various opportunistic infections [1–4, 11, 21]. Several studies have proposed that the immunodeficiency involves the neutralization of IFN-γ activity by the autoantibody [1–4, 11, 21]. We investigated other immune parameters which may also contribute to this immunodeficiency.

Increasing numbers of white blood cells and phagocytes in the general circulation is used as a marker of infection. A trend towards higher numbers of white blood cells and phagocytes was observed in patients with anti-IFN-γ AAbs. These results correlate with the C-reactive protein (CRP) level in patients’ sera. In this study, 48% of the enrolled patients have higher CRP level from the normal range. In the patients with and without active opportunistic infection during the past 30 days, 75% and 38% had higher CRP level from the normal range, respectively. The increased CRP and WBC levels indicate the presence of inflammation in these patients. This indicates that the immunodeficiency symptom and repeat infection in patients with autoantibodies against IFN-γ is not due to panleukopenia or a reduction in the number of phagocytes. The NK cell population also increased in the patients, indicating an alteration in the NK cells in patients with and without active opportunistic infection during the past 30 days (data not shown). The chronic and repeating opportunistic infections occurring in patients with anti-IFN-γ AAbs may activate and cause an increase in the number of NK cells. Incidentally, as IFN-γ is considered the prototypic NK cell cytokine, the diminished activity of IFN-γ due to the neutralizing anti-IFN-γ AAbs in the patients may induce a compensatory effect by increasing the NK cell number.

Cell-mediated immunity (CMI) plays a central role in the eradication of infectious diseases [20, 22, 23]. This type of immunity is dependent on the recognition of antigen by TCR expressed on the T cell surface and their subsequent destruction of cells bearing the antigen, or on the secretion of lymphokines that enhance the ability of phagocytes to eliminate the invaded microbes, particularly intracellular microbes [23]. Patients with anti-IFN-γ AAbs have been reported to be susceptible to various types of intracellular infections, for example, disseminated non-tuberculous mycobacterial infection, disseminated penicilliosis marneffei, non-typhoidal Salmonella bacteremia, cytococcosis, histoplasmosis, and disseminated herpes zoster, with a relative high mortality rate [1–4, 11, 21]. Autoantibody to IFN-γ was proposed to be the cause of CMI immunodeficiency. We speculate that, besides the presence of the anti-IFN-γ antibody, T cell responses may be dysfunctional in these patients. The activation of TCR on T cells was carried out using anti-CD3 mAb. Upon TCR activation, the T cell proliferation and the CD25 (IL-2 receptor) expression of the patients with anti-IFN-γ AAbs remained intact. The results indicate that at least part of the signal transduction subsequence of TCR triggering was still intact. As cytokines produced by T cells are the crucial mediators for cell-mediated immunity [23], we further determined the T helper (Th) cell cytokines after T cell activation. Surprisingly, after PMA and ionomycin stimulation, the Th1 cytokine production, including the production of IFN-γ, and TNF-α, were up-regulated in patients with anti-IFN-γ AAbs, in comparison with the healthy subjects. Our results were not in agreement with a previous report which observed a reduction in the production of TNF-α and IL-2 [12]. Compared to this previous report [12], in our study, PMA and ionomycin were used as stimulators, instead of PHA. PHA and PMA/ionomycin act on cells in a different manner. By using PHA activation, the signals could be coming from any glycosylated surface molecule. In contrast, PMA directly activates protein kinase C (PKC) omitting the requirement of surface receptor stimulation. Ionomycin, a calcium ionophor, is able to trigger calcium release which is required for NFAT signaling. The PMA/ionophor activation is, therefore, closer to the physiological condition for T cell activation [24]. In this study, although enhancement of IFN-γ and TNF-α production was observed in the patients, the production of Th2 (IL4) and Th17 (IL-17) cytokines were at the same level as healthy controls. To confirm whether enhancement of Th1 cytokines occurred under physiological conditions, activation of TCR by anti-CD3 mAb was performed. Up-regulation of IFN-γ production in the patients with anti-IFN-γ AAbs was observed. Our results indicate an alteration in cell-mediated immune response in the patients with anti-IFN-γ AAbs.

In this study, the patients enrolled seem to be older than the healthy subjects. Reduction of T proliferation, IL-2 production and IL-2 receptor expression have been reported in the elderly [25–27]. However, the age of affect was observed in patients with an average at 65 or older [26–28], which was much older than our patient group. In addition, we found no statistically significant difference between patients and healthy subjects in T cell proliferation and the expression of CD25 (IL-2 receptor) as shown in S1 and S2 Figs. The observed increase of IFN-γ and TNF-α production in T cells may be related to an alteration of the Th1/Th2 balance in patients with anti-IFN-γ AAbs. The mechanism of the up-regulation of IFN-γ and TNF-α in the patients is, however, unclear. We speculate that T cells of the patients have already committed to be Th1 cells. The imbalance of Th1 and Th2 cytokine has been reported in some autoimmune diseases. Th1 dominant was observed in autoimmune disease such as multiple sclerosis (MS), inflammatory bowel diseases (IBD), Rheumatoid Arthritis (RA) and Hashimoto’s Thyroiditis [29]. These autoimmune diseases have high levels of IFN-γ and uncontrolled inflammation and infection [30, 31]. Based on our findings, we proposed that there may be a defect in the regulatory function of the immune system resulting in an error to control the Th1 and Th2 balance in patients with anti-IFN-γ AAbs. Excessive Th1 cytokines, i.e. IFN-γ, have been reported to induce autoimmune disease [30, 31]. We hypothesize that the over production of Th1 cytokines observed in these patients may alter the controlling of auto-reactive B cells results in the production of anti-IFN-γ AAbs. The overwhelmed Th1 activation and the enhanced Th1 cytokine production in these patients may also be due to a compensation mechanism in which the ability of IFN-γ was neutralized by the autoantibodies. It is also possible that activation of T cells *in vivo* following chronic antigenic stimulation may also have occurred and may need to be taken into consideration.

## Supporting Information

S1 Fig T cell proliferation in patient with anti-IFN-γ AAbs and healthy subjects. CFSE-labeled PBMCs were stimulated with immobilized anti-CD3 mAb. The T cell proliferation of the patients with anti-IFN-γ AAbs and the healthy subjects upon anti-CD3 activation was presented as percentage of divided cells in the dot density plot.
(TIF)

S2 Fig CD25 expressions in patients with anti-IFN-γ AAbs and healthy subjects. PBMCs were stimulated or un-stimulated with immobilized anti-CD3 mAb. (A) The expression of CD25 was found to have increased in all the tested groups after stimulation. The stimulated cells are presented in close gray histogram plots and the un-stimulated cells of each sample are overlaid in open black histogram plots. (B) The expressions of CD25 in the patient with anti-IFN-γ AAbs and the healthy subjects are presented as the ratio of mean fluorescent intensity (MFI) of activation and no activation in the dot density plot. (C) The percentage of the CD25 expressing cells is presented, and it was observed that there was no difference between the tested groups. The bars represent the mean of the percentages of the CD25 expressing cells. The error bars indicate the SD value.
(TIF)

## References

[1] Browne SK, Burbelo PD, Chetchotisakd P, Suputtamongkol Y, Kiertiburanakul S, Shaw PA, et al Adult-onset immunodeficiency in Thailand and Taiwan. N Engl J Med. 2012;367:725–34. 10.1056/NEJMoa1111160 . 22913682 PMC4190026

[2] Chi CY, Chu CC, Liu JP, Lin CH, Ho MW, Lo WJ, et al Anti-IFN-gamma autoantibodies in adults with disseminated nontuberculous mycobacterial infections are associated with HLA-DRB1*16:02 and HLA-DQB1*05:02 and the reactivation of latent varicella-zoster virus infection. Blood. 2013;121:1357–66. 10.1182/blood-2012-08-452482 . 23243276

[3] Koya T, Tsubata C, Kagamu H, Koyama K, Hayashi M, Kuwabara K, et al Anti-interferon-gamma autoantibody in a patient with disseminated Mycobacterium avium complex. J Infect Chemother. 2009;15:118–22. 10.1007/s10156-008-0662-8 . 19396523

[4] Wongkulab P, Wipasa J, Chaiwarith R, Supparatpinyo K. Autoantibody to interferon-gamma associated with adult-onset immunodeficiency in non-HIV individuals in Northern Thailand. PLoS One. 2013;8:e76371 10.1371/journal.pone.0076371 . 24086734 PMC3785451

[5] Browne SK, Holland SM. Immunodeficiency secondary to anticytokine autoantibodies. Curr Opin Allergy Clin Immunol. 2010;10:534–41. . 20966748 10.1097/ACI.0b013e3283402b41 PMC3132574

[6] Browne SK. Anticytokine autoantibody-associated immunodeficiency. Annu Rev Immunol. 2014;32:635–57. 10.1146/annurev-immunol-032713-120222 . 24499273

[7] Prummer O, Bunjes D, Wiesneth M, Arnold R, Porzsolt F, Heimpel H. High-titre interferon-alpha antibodies in a patient with chronic graft-versus-host disease after allogeneic bone marrow transplantation. Bone Marrow Transplant. 1994;14:483–6. . 7994279

[8] Rosen SH, Castleman B, Liebow AA. Pulmonary alveolar proteinosis. N Engl J Med. 1958;258:1123–42. 10.1056/NEJM195806052582301 . 13552931

[9] Meager A, Wadhwa M, Dilger P, Bird C, Thorpe R, Newsom-Davis J, et al Anti-cytokine autoantibodies in autoimmunity: preponderance of neutralizing autoantibodies against interferon-alpha, interferon-omega and interleukin-12 in patients with thymoma and/or myasthenia gravis. Clin Exp Immunol. 2003;132:128–36. . 12653847 10.1046/j.1365-2249.2003.02113.x PMC1808678

[10] Kisand K, Boe Wolff AS, Podkrajsek KT, Tserel L, Link M, Kisand KV, et al Chronic mucocutaneous candidiasis in APECED or thymoma patients correlates with autoimmunity to Th17-associated cytokines. J Exp Med. 2010;207:299–308. 10.1084/jem.20091669 . 20123959 PMC2822605

[11] Poulin S, Corbeil C, Nguyen M, St-Denis A, Cote L, Le Deist F, et al Fatal Mycobacterium colombiense/cytomegalovirus coinfection associated with acquired immunodeficiency due to autoantibodies against interferon gamma: a case report. BMC Infect Dis. 2013;13:24 10.1186/1471-2334-13-24 . 23336346 PMC3561114

[12] Wipasa J, Wongkulab P, Chawansuntati K, Chaiwarit R, Supparatpinyo K. Cellular immune responses in HIV-negative immunodeficiency with anti-interferon-gamma antibodies and opportunistic intracellular microorganisms. PLoS One. 2014;9:e110276 10.1371/journal.pone.0110276 . 25329064 PMC4203775

[13] Schoenborn JR, Wilson CB. Regulation of interferon-gamma during innate and adaptive immune responses. Adv Immunol. 2007;96:41–101. 10.1016/S0065-2776(07)96002-2 . 17981204

[14] Schroder K, Hertzog PJ, Ravasi T, Hume DA. Interferon-gamma: an overview of signals, mechanisms and functions. J Leukoc Biol. 2004;75:163–89. 10.1189/jlb.0603252 . 14525967

[15] Billiau A, Matthys P. Interferon-gamma: a historical perspective. Cytokine Growth Factor Rev. 2009;20:97–113. 10.1016/j.cytogfr.2009.02.004 . 19268625

[16] Abbas AK, Lichtman AH, Pillai S. Cellular and molecular immunology. 6th ed Philadelphia, Pa.: Saunders Elsevier; 2010 p. 3–17.

[17] Doffinger R, Patel S, Kumararatne DS. Human immunodeficiencies that predispose to intracellular bacterial infections. Curr Opin Rheumatol. 2005;17:440–6. . 15956841 10.1097/01.bor.0000166387.70475.dd

[18] Al-Muhsen S, Casanova JL. The genetic heterogeneity of mendelian susceptibility to mycobacterial diseases. J Allergy Clin Immunol. 2008;122:1043–51. 10.1016/j.jaci.2008.10.037 . 19084105

[19] Crum NF, Lederman ER, Wallace MR. Infections associated with tumor necrosis factor-alpha antagonists. Medicine (Baltimore). 2005;84:291–302. . 16148729 10.1097/01.md.0000180044.19285.9a

[20] Haverkamp MH, van Dissel JT, Holland SM. Human host genetic factors in nontuberculous mycobacterial infection: lessons from single gene disorders affecting innate and adaptive immunity and lessons from molecular defects in interferon-gamma-dependent signaling. Microbes Infect. 2006;8:1157–66. 10.1016/j.micinf.2005.10.029 . 16520075

[21] Tang BS, Chan JF, Chen M, Tsang OT, Mok MY, Lai RW, et al Disseminated penicilliosis, recurrent bacteremic nontyphoidal salmonellosis, and burkholderiosis associated with acquired immunodeficiency due to autoantibody against gamma interferon. Clin Vaccine Immunol. 2010;17:1132–8. 10.1128/CVI.00053-10 . 20445006 PMC2897261

[22] van de Vosse E, Hoeve MA, Ottenhoff TH. Human genetics of intracellular infectious diseases: molecular and cellular immunity against mycobacteria and salmonellae. Lancet Infect Dis. 2004;4:739–49. 10.1016/S1473-3099(04)01203-4 . 15567123

[23] Abbas AK, Lichtman AH, Pillai S. Cellular and molecular immunology. 7th ed Philadelphia, Pa.: Saunders Elsevier; 2012 p. 225.

[24] Chopra RK, Powers DC, Adler WH, Nagel JE. Phorbol myristate acetate and calcium ionophore A23187-stimulated human T cells do not express high-affinity IL-2 receptors. Immunology. 1989;66:54–60. . 15493263 PMC1385120

[25] Desai A, Grolleau-Julius A, Yung R. Leukocyte function in the aging immune system. J Leukoc Biol. 2010;87:1001–9. 10.1189/jlb.0809542 . 20200405 PMC4057658

[26] Nagel JE, Chopra RK, Chrest FJ, McCoy MT, Schneider EL, Holbrook NJ, et al Decreased proliferation, interleukin 2 synthesis, and interleukin 2 receptor expression are accompanied by decreased mRNA expression in phytohemagglutinin-stimulated cells from elderly donors. J Clin Invest. 1988;81:1096–102. 10.1172/JCI113422 . 3127423 PMC329636

[27] Nagel JE, Chopra RK, Powers DC, Adler WH. Effect of age on the human high affinity interleukin 2 receptor of phytohaemagglutinin stimulated peripheral blood lymphocytes. Clin Exp Immunol. 1989;75:286–91. . 2784739 PMC1542135

[28] Whisler RL, Beiqing L, Chen M. Age-related decreases in IL-2 production by human T cells are associated with impaired activation of nuclear transcriptional factors AP-1 and NF-AT. Cell Immunol. 1996;169:185–95. 10.1006/cimm.1996.0109 . 8620546

[29] Damsker JM, Hansen AM, Caspi RR. Th1 and Th17 cells: adversaries and collaborators. Ann N Y Acad Sci. 2010;1183:211–21. 10.1111/j.1749-6632.2009.05133.x . 20146717 PMC2914500

[30] Trinchieri G. Type I interferon: friend or foe? J Exp Med. 2010;207:2053–63. 10.1084/jem.20101664 . 20837696 PMC2947062

[31] Pollard KM, Cauvi DM, Toomey CB, Morris KV, Kono DH. Interferon-gamma and systemic autoimmunity. Discov Med. 2013;16:123–31. . 23998448 PMC3934799

