# Supplementary figures and images for: Immune Alterations in Patients with Anti-Interferon-γ Autoantibodies
Source: PLoS One. 2016 Jan 4;11(1):e0145983. doi: 10.1371/journal.pone.0145983 (PMC4699769; doi:10.1371/journal.pone.0145983)

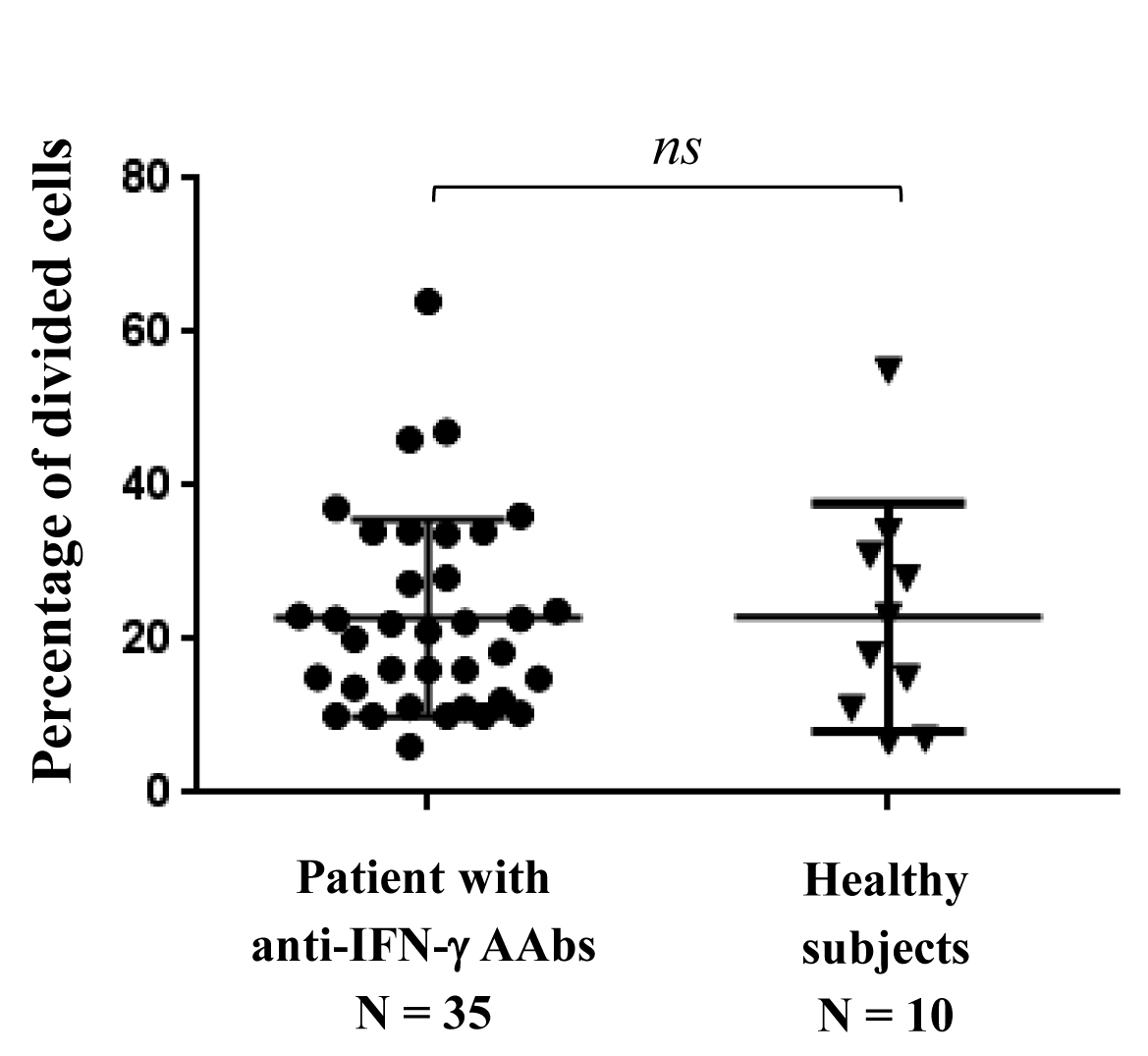

Supplement: S1 Fig — CFSE-labeled PBMCs were stimulated with immobilized anti-CD3 mAb. The T cell proliferation of the patients with anti-IFN-γ AAbs and the healthy subjects upon anti-CD3 activation was presented as percentage of divided cells in the dot density plot. (TIF) [file pone.0145983.s001.tif]

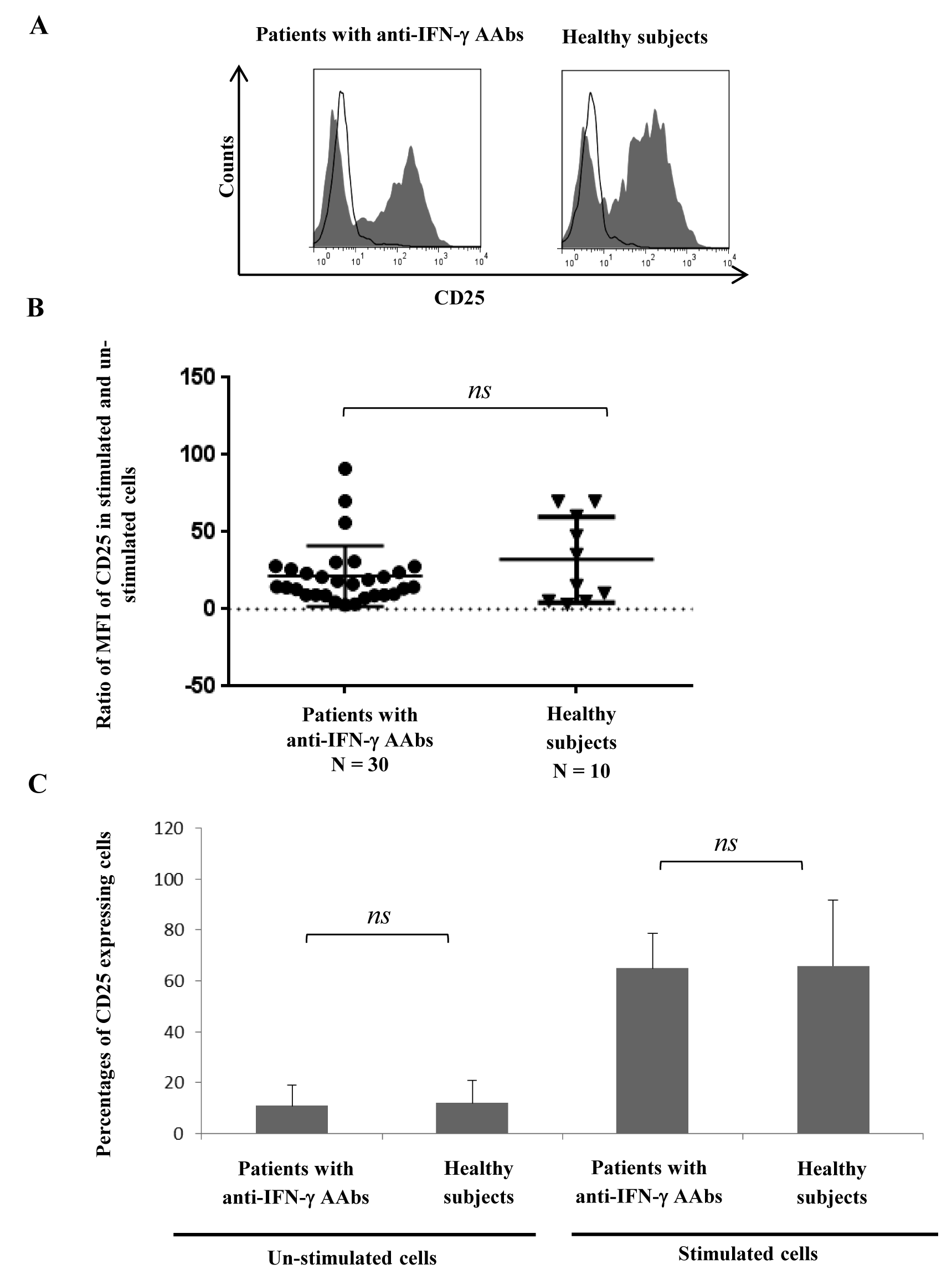

Supplement: S2 Fig — PBMCs were stimulated or un-stimulated with immobilized anti-CD3 mAb. (A) The expression of CD25 was found to have increased in all the tested groups after stimulation. The stimulated cells are presented in close gray histogram plots and the un-stimulated cells of each sample are overlaid in open black histogram plots. (B) The expressions of CD25 in the patient with anti-IFN-γ AAbs and the healthy subjects are presented as the ratio of mean fluorescent intensity (MFI) of activation and no activation in the dot density plot. (C) The percentage of the CD25 expressing cells is presented, and it was observed that there was no difference between the tested groups. The bars represent the mean of the percentages of the CD25 expressing cells. The error bars indicate the SD value. (TIF) [file pone.0145983.s002.tif]
